# Retraction Note: Empirical Modeling of Physiochemical Immune Response of Multilayer Zinc Oxide Nanomaterials under UV Exposure to Melanoma and Foreskin Fibroblasts

**DOI:** 10.1038/s41598-023-40251-0

**Published:** 2023-08-09

**Authors:** Muhammad Fakhar-e-Alam, M. Waseem Akram, Seemab Iqbal, K. S. Alimgeer, M. Atif, K. Sultana, M. Willander, Zhiming M. Wang

**Affiliations:** 1 Institute of Fundamental and Frontier Science, University of Electronic Science and Technology of China, 610054 Chengdu, China; 2 Department of Science and Technology, Campus Norrköping, Linköping University, SE-601 74 Norrköping, Sweden; 3 Department of Physics, GC University, 38000 Faisalabad, Pakistan; 4 COMSATS Institute of Information Technology, Islamabad, Pakistan; 5 Department of Physics and Astronomy, College of Science, King Saud University, Riyadh, Saudi Arabia; 6 National Institute of Laser and Optronics, Nilore, Islamabad, Pakistan

Retraction of: *Scientific Reports* 10.1038/srep46603, published online 24 April 2017

The Editors have retracted this Article.

Figure 1 appears to be in part identical to Figure 1 of another published article^1^, despite the materials and methods describing how the nanorods were grown differently. The fluorescent spectrum presented in Figure 6 also appears to be identical to Figure 2B of^1^, and Figure 2 of^2^. The image for Figure 2C appears to have been duplicated in Figure 7C. This image also appears to have been published as Figure 3D in^2^, and Figure 12C of^3^. The images for Figure 7B and 7D appear to have been published, respectively, as Figure 12B and 12D in^3^. The Editors therefore no longer have confidence in the validity of the data and the conclusions drawn.

M. Waseem Akram and Zhiming M. Wang agree with the retraction and its wording. Muhammad Fakhar-e-Alam, Seemab Iqbal, K. S. Alimgeer, M. Atif, and M. Willander did not respond to the correspondence about retraction. The Editors were not able to establish current contact details for K. Sultana.
